# Molecular Analysis of Canine Filaria and Its *Wolbachia* Endosymbionts in Domestic Dogs Collected from Two Animal University Hospitals in Bangkok Metropolitan Region, Thailand

**DOI:** 10.3390/pathogens8030114

**Published:** 2019-07-29

**Authors:** Hathaithip Satjawongvanit, Atchara Phumee, Sonthaya Tiawsirisup, Sivapong Sungpradit, Narisa Brownell, Padet Siriyasatien, Kanok Preativatanyou

**Affiliations:** 1 Medical Science Program, Faculty of Medicine, Chulalongkorn University, Bangkok 10330, Thailand; 2 Vector Biology and Vector Borne Disease Research Unit, Department of Parasitology, Faculty of Medicine, Chulalongkorn University, Bangkok 10330, Thailand; 3 Thai Red Cross Emerging Infectious Disease-Health Science Centre, World Health Organization Collaborating Centre for Research and Training on Viral Zoonoses, Chulalongkorn Hospital, Bangkok 10330, Thailand; 4 Veterinary Parasitology Unit, Department of Veterinary Pathology, Faculty of Veterinary Science, Chulalongkorn University, Bangkok 10330, Thailand; 5 Department of Pre-clinic and Applied Animal Science, Faculty of Veterinary Science, Mahidol University, Salaya, Nakhon Pathom 73170, Thailand

**Keywords:** dog, filariasis, *D. immitis*, *B. pahangi*, *B. malayi*, zoonosis, Thailand

## Abstract

Canine filariasis is caused by several nematode species, such as *Dirofilaria immitis*, *Dirofilaria repens*, *Brugia pahangi*, *Brugia malayi*, and *Acanthocheilonema reconditum*. Zoonotic filariasis is one of the world’s neglected tropical diseases. Since 2000, the World Health Organization (WHO) has promoted a global filarial eradication program to eliminate filariasis by 2020. Apart from vector control strategies, the infection control of reservoir hosts is necessary for more effective filariasis control. In addition, many studies have reported that *Wolbachia* is necessary for the development, reproduction, and survival of the filarial nematode. Consequently, the use of antibiotics to kill *Wolbachia* in nematodes has now become an alternative strategy to control filariasis. Previously, a case of subconjunctival dirofilariasis caused by *Dirofilaria* spp. has been reported in a woman who resides in the center of Bangkok, Thailand. Therefore, our study aimed to principally demonstrate the presence of filarial nematodes and *Wolbachia* bacteria in blood collected from domestic dogs from the Bangkok Metropolitan Region, Thailand. A total of 57 blood samples from dogs with suspected dirofilariasis who had visited veterinary clinics in Bangkok were collected. The investigations for the presence of microfilaria were carried out by using both microscopic and molecular examinations. PCR was used as the molecular detection method for the filarial nematodes based on the *COI* and *ITS1* regions. The demonstration of *Wolbachia* was performed using PCR to amplify the *FtsZ* gene. All positive samples by PCR were then cloned and sequenced. The results showed that the filarial nematodes were detected in 16 samples (28.07%) using microscopic examinations. The molecular detection of filarial species using *COI*-PCR revealed that 50 samples (87.72%) were positive; these consisted of 33 (57.89%), 13 (22.81%), and 4 (7.02%) samples for *D. immitis*, *B. pahangi*, and *B. malayi,* respectively. While the *ITS1*-PCR showed that 41 samples (71.93%) were positive—30 samples (52.63%) were identified as containing *D. immitis* and 11 samples (19.30%) were identified to have *B. pahangi*, whereas *B. malayi* was not detected. Forty-seven samples (82.45%) were positive for *Wolbachia* DNA and the phylogenetic tree of all positive *Wolbachia* was classified into the supergroup C clade. This study has established fundamental data on filariasis associated with *Wolbachia* infection in domestic dogs in the Bangkok Metropolitan Region. An extensive survey of dog blood samples would provide valuable epidemiologic data on potential zoonotic filariasis in Thailand. In addition, this information could be used for the future development of more effective prevention and control strategies for canine filariasis in Thailand.

## 1. Introduction

Filariasis is a mosquito-borne parasitic disease, which is an important public health concern in tropical and subtropical areas. It affects approximately 886 million people in 52 countries worldwide [1]. The disease is commonly found in Southeast Asian countries [2]. Filariasis has been reported as an important zoonosis from dogs and cats, both of which are well recognized as reservoirs for filariasis [3]. Several major canine filarial species have been documented in several localities around the world, including *Dirofilaria immitis*, *Dirofilaria repens*, *Brugia malayi*, *Brugia pahangi*, *Brugia ceylonensis*, *Brugia patei*, *Cercopithifilaria grassii*, *Acanthocheilonema reconditum*, and *Acanthocheilonema dracunculoides* [4,5,6]. In Thailand, the common filarial worms in dogs are *D. immitis*, *B. malayi*, *B. pahangi,* and *A. reconditum* [7,8,9]. *D. immitis* is the most common species; it causes canine heartworm disease or canine dirofilariasis and is widespread. The disease is endemic in tropical and subtropical regions throughout the world [10]. Among the neglected tropical diseases, filariasis was selected as a particular target for achieving elimination by 2020, and this has been published in the World Health Organization (WHO) roadmap [11,12]. Previously, the elimination and control strategy of filariasis was based on the control of the mosquito vectors, such as those in the *Mansonia*, *Anopheles*, *Aedes, Culex,* and *Ochlerotatus* genuses [13,14]. However, animals, especially dogs, can also serve as the reservoirs of filarial nematodes. Therefore, the control of infection in all potential reservoirs is also essential. Moreover, a study in dog reservoirs might provide a more in-depth understanding of the patterns and epidemiologic data of filariasis, and it could also contribute to a decrease in the number of human cases. Zoonotic filariasis has been reported globally. In Thailand, several human cases of zoonotic filariasis have been documented. Some examples include the ocular *Dirofilaria* infections caused by *Dirofilaria* spp. [15] and *D. repens* [16] in the patients from Phangnga and Nakhon Si Thammarat provinces, respectively. The most recently reported case was in 2018. A 67-year-old woman residing in Bangkok presented with subconjunctival dirofilariasis. Molecular analysis of the sample obtained from the eye of the patient showed that it was caused by an unknown *Dirofilaria* spp. closely related to *Dirofilaria hongkongensis* [17]. 

Several reports suggest that filarial nematodes serve as hosts for an obligate bacterial endosymbiont, *Wolbachia* [18,19,20]. *Wolbachia,* a gram-negative intracellular bacterium, plays an important role in nematode biological development, reproduction, and survival. It also provides critical metabolites to the filarial nematode [11,21,22]. In addition, the correlation between *Wolbachia*–nematode symbiosis has provided treatment strategies for the control and eradication of the filarial infection in the host using *Wolbachia* as a target of antibiotic treatment [23,24]. Many studies show that filarial nematode in dogs can be diagnosed through the examination of circulating microfilariae [25,26,27]. However, when the Giemsa stain is used for characterization, it is difficult to differentiate and identify definite species among the closely related microfilaria species because of the similarities in their morphology. As previously mentioned, there is a report on the zoonotic case in Bangkok caused by an unknown *Dirofilaria* species, and the morphology identification problem of the blood stage nematode and the relationship between nematodes and *Wolbachia* remain unsolved. Therefore, the aims of our study are primarily to determine the presence of filaria nematodes in domestic dog blood samples from the Bangkok Metropolitan Region, Thailand using molecular techniques to precisely identify the species of detected filarial nematodes. Polymerase chain reaction (PCR) was used as a molecular technique to amplify the cytochrome c oxidase subunit I (*COI*) gene and internal transcribed spacer 1 (*ITS1*) region, which are both suitable molecular markers given the high degree of genetic variation in filarial nematodes. The study was also used to investigate the status of *Wolbachia* symbionts in filarial nematodes in Thailand. The presence of *Wolbachia* bacteria was demonstrated by PCR to amplify the filamenting temperature-sensitive protein mutant Z (*FtsZ*) gene, which plays a role in the cell division of *Wolbachia* and can also be found in high copy numbers. Sequences obtained from the study were used for phylogenetic tree construction. Information obtained from the study provides preliminary data on filariasis associated with *Wolbachia* infection in domestic dogs in Bangkok, Thailand. These data will be useful in the future for reservoir- and *Wolbachia*-based programs for filariasis control.

## 2. Results

### 2.1. Morphological Characteristics of Filarial Worm

**Figure 1:**
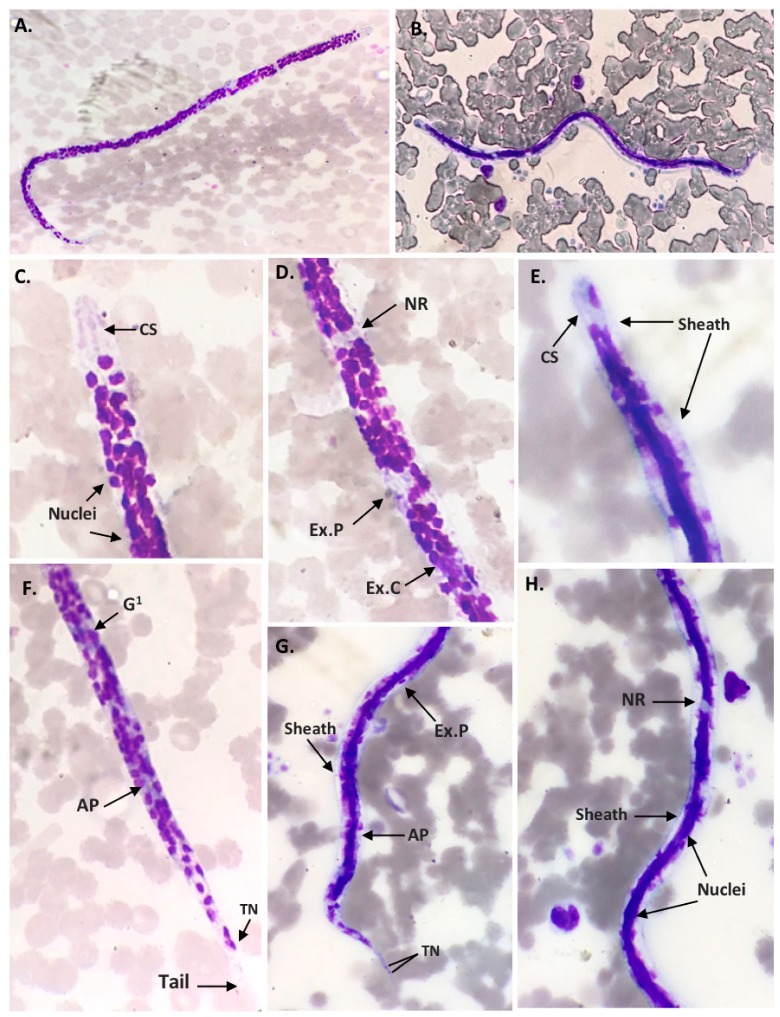
Microfilaria of *Dirofilaria* spp. (**A**,**C**,**D**,**F**) and *Brugia* spp. (**B**,**E**,**G**,**H**), Giemsa stain, 100×. The morphological marks show the position of several structures: The cephalic space (CS), nerve ring (NR), excretory pore (Ex.P), excretory cell (Ex.C), genital cell (G1), anal pore (AP), and terminal nucleus (TN).

Microfilariae were detected in 16 of 57 (28.07%) dog blood samples collected in the Bangkok Metropolitan Region using the Giemsa staining technique. The morphological identification for microfilariae species found that 11 samples (19.30%) were *Dirofilaria* spp. and 5 samples (8.77%) were *Brugia* spp. (Table 1). For the *Dirofilaria* spp., Giemsa staining showed unsheathed microfilaria and the relative positions of the nerve ring (NR), excretory pore (Ex.P), excretory cell (Ex.C), first genital cell (G1), anal pore (AP), and the terminal nucleus (TN) at the tail. (Figure 1). For the *Brugia* spp., Giemsa staining showed clear sheathed microfilaria and one nucleus in the elongated cephalic space. The position of the nerve ring (NR), excretory pore (Ex.P), anal pore (AP), and two discrete overlapping terminal nuclei at the tail end are shown in Figure 1.

### 2.2. Molecular Detection of Filarial Nematode

**Figure 2:**
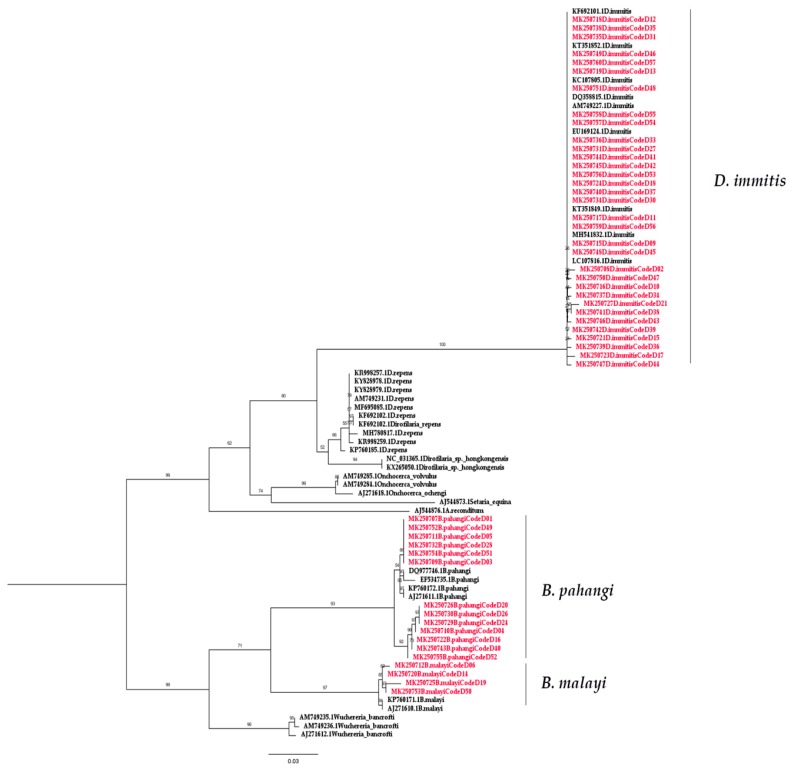
Phylogenetic tree of filarial worms constructed from the partial *COI* sequences. The red color represents the individual and combination identification sequences compared with reference isolates obtained from GenBank. Branch support was estimated based on 1000 bootstrap replicates.

**Figure 3:**
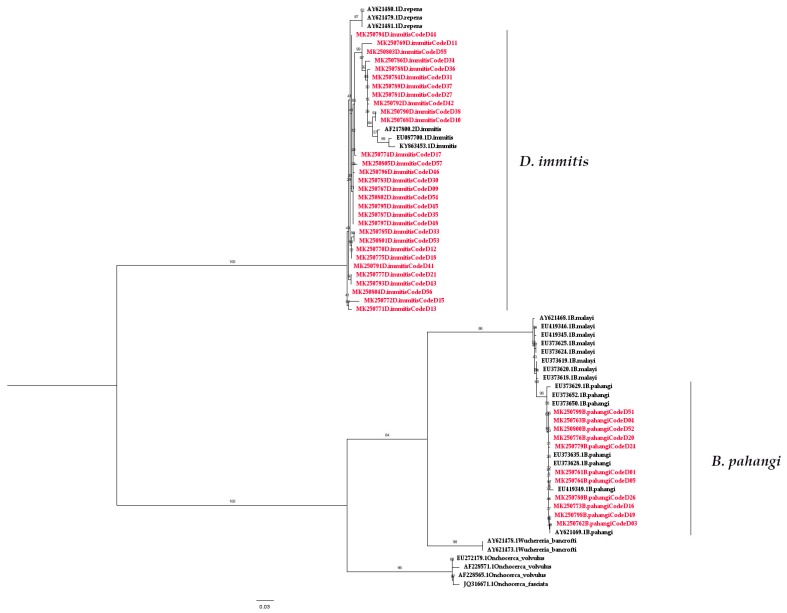
Phylogenetic tree of filarial worms constructed from *ITS1* sequences. The red color represents the individual and combination identification sequences compared with reference isolates obtained from GenBank. Branch support was estimated based on 1000 bootstrap replicates.

Both *COI*-PCR and *ITS1*-PCR were able to detect filarial nematode DNA in the blood samples. Of the 57 blood samples collected, 50 (87.72%) samples were amplified. The nucleotide sequences of the partial *COI* gene contained approximately 690 bp (Figure S1) and were able to classify filarial nematodes into three species: *D. immitis, B. pahangi,* and *B. malayi,* accounting for 33 (57.89%), 13 (22.81%), and 4 (7.02%) of the samples, respectively. Meanwhile, 41 samples (71.93%) were amplified for filarial nematode DNA using PCR annealed specifically to the *ITS1* region, which has approximately 600 bp for *D. immitis* (52.63%) and 550 bp for *B. pahangi* (19.30%) (Table 1, Figure S2). According to the sequence analysis of all sequences included in this study (Figures S3 and S4), the results of partial *COI* sequence comparisons between the species showed a sequence similarity percentage of 98.9%–100% (mean of 99.72%), 98.2%–100% (mean of 99.05%), and 98.6%–100% (mean of 99.22%) for *D. immitis*, *B. pahangi,* and *B. malayi* respectively, whereas the sequence similarity of *ITS1* was 83.3%–100% (mean of 92.2%) and 96.3%–100% (mean of 98.3%) for *D. immitis* and *B. pahangi,* respectively. The phylogenetic tree based on the partial *COI* and *ITS1* sequence showed that all isolated samples from *D. immitis* clustered together in one group. Two isolated from *B. pahangi* and *B. malayi* were clustered together, and they were clearly separated branches despite being placed in the same genus (Figure 2 and Figure 3). The partial nucleotide sequences of the *COI* and *ITS1* obtained in this study were deposited in the GenBank under accession numbers MK250707–MK250757 and MK250758–MK250799, respectively. Based on the results, *COI*-PCR could be better suited for filarial species identification than *ITS1*-PCR. Additionally, present studies showed that *COI* evolves much more quickly than *ITS1*, revealing low intraspecific and interspecific molecular variability for filarial worms.

### 2.3. Wolbachia Detection Using Nested PCR

**Figure 4:**
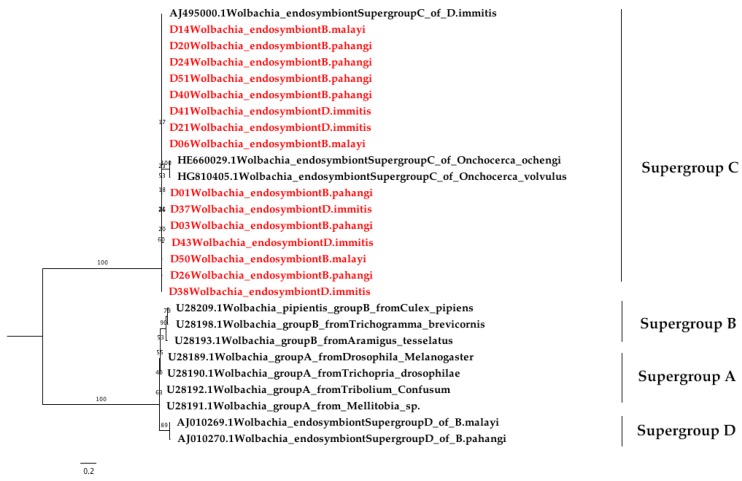
Phylogenetic tree of *Wolbachia* from filarial worms constructed from the partial *FtsZ* sequences. The red color represents the individual and combination identification sequences compared with reference isolates obtained from GenBank. Branch support was estimated based on 1000 bootstrap replicates.

*Wolbachia* DNA was detected based on the *Wolbachia* protein-coding housekeeping *FtsZ* gene (147 bp) (Figure S5). The result showed that 47 of 57 (82.45%) blood dog samples were positive for *Wolbachia* DNA. The species detected consisted of *D. immitis* (57.89%), *B. pahangi* (19.30%), and *B. malayi* (5.26%) (Table 2). The nucleotide sequence of *FtsZ* of the *Wolbachia* endosymbiont (Figure S6) had a similarity of 94.5%–100% (mean of 99.64%). The phylogenetic tree based on the *FtsZ* gene revealed that 47 of the *Wolbachia* DNA samples in this study could be clearly classified into supergroup C (Figure 4).

## 3. Discussion

Canine filarioids are important nematodes that are transmitted to vertebrate hosts through arthropods and mosquitoes. Nematodes can cause severe disease in dogs and, potentially, transmit the disease to humans [28]. Control of canine filariasis through vector control and the early treatment of infected reservoir hosts is essential. Therefore, a highly specific and sensitive technique for the diagnosis of canine filarioids from an early stage of infection is necessary. Although the gold standard method for the diagnosis of microfilariae in a blood smear is microscopic examination on glass slides stained with Giemsa or hematoxylin and eosin [29,30], this technique requires considerable expertise, has low sensitivity, and cannot clearly discriminate among closely related species of filarial nematodes, such as *D. immitis*, *D. repens,* and *D. reconditum* or *B. malayi* and *B. pahangi* [31]. Serologic and molecular techniques with high sensitivity and specificity for filariasis diagnosis have been developed as additional methods for the detection of microfilariae [6,32,33,34]. In this study, microscopic examination gives a low positive result for microfilariae (28.07%) compared to PCR. We investigated conventional PCR for both canine filarial and *Wolbachia* bacterial infections in dog blood samples. PCR could be used to increase the detection rate of microfilaria DNA and also to differentiate filarial parasites among closely related species. This study also suggests that *COI* sequences can act as useful genetic markers in distinguishing between *D. immitis*, *B. pahangi,* and *B. malayi*; however, *ITS1* can detect only *D. immitis* and *B. pahangi*. Thus, the *COI*-PCR method is a more suitable method for the PCR-based detection of filarial nematode infections in dog blood specimens than *ITS1*-PCR. Therefore, we recommend using the *COI* gene for filarial detection because it is commonly used in the study of filarial DNA detection and has shown a large number of homolog sequences available in GenBank for the comparison and evaluation of results. Unfortunately, the limitation of this study was that the number of dog blood samples was quite low in number. To increase the accuracy of these assays, a larger sample size might be needed. 

There are several reports on the prevalence of major *D. immitis* infection in dogs from various countries, including China [35], Korea [36], Iran [37], Portugal [38], Hungary [39], and Thailand [7,8,9,40]. In this current study, we also found that *D. immitis* is the most frequent species found in dog blood samples using both *COI*-PCR (57.89%) and *ITS1*-PCR (52.63%). This is followed by *B. pahangi,* detected in 22.81% of samples using *COI*-PCR and 19.30% of samples using *ITS1*-PCR. In contrast, *B. malayi* can be detected in only 7.02% of samples using *COI*-PCR. According to previous studies, both *B. malayi* and *B. pahangi* infections in dogs [41,42,43] and cats [7,9,44] have been found in different regions of southern Thailand, such as the Narathiwat and Satun provinces [7,8,9]. However, the information on filariasis in dogs caused by *B. malayi* and *B. pahangi* in terms of their relationship with their hosts, geographic distribution, and association with human or veterinary diseases is quite limited. Our study showed that the blood samples from dogs in the Bangkok Metropolitan Region were infected with both *B. malayi* and *B. pahangi*. Therefore, we suspect that domestic dogs may be a potential reservoir for *B. malayi* and *B. pahangi* in Bangkok. Further surveys throughout the country to investigate the prevalence of *B. malayi* and *B. pahangi* infection in a greater number of dog samples are needed in order to investigate the exact role of dogs as the natural reservoir hosts of *B. malayi* and *B. pahangi*. Further investigations on the mosquito vectors are also needed for the development of control protocols in the future. 

The preliminary study of the phylogenetic tree based on the partial *COI* sequences of *B. malayi–*positive samples showed they were clustered together with *B. malayi* from France (accession no. KP760171.1) and Italy (accession no. AJ271610.1) with short branch lengths. The *B. pahangi* sequences of *COI* were also clustered together with *B. pahangi* from Malaysia (accession no. DQ977746.1 and EF534735.1), France (accession no. KP760172.1), and Italy (accession no. AJ271611.1) with small branch lengths. This is also true for *B. pahangi* sequences based on the *ITS1* region. According to the result of the phylogenetic tree of filarial worms constructed from partial *COI* sequences, two isolated samples from *B. malayi* and *B. pahangi* were in the same cluster and could be clearly separated. For this reason, the *COI* sequence was useful for identifying these congeneric species, and *COI*-PCR also showed better amplification for multiple species than *ITS1*-PCR in the present study. Oh et al. (2017) suggested that *COI* is the so-called “barcode” for the identification of filarial species diversity [45]. The strength of this study is that this method can be used as a screening and survey tool to study the molecular epidemiology of filarial infection among the high-risk population. In this study, we did not find *D. hongkongensis* as we expected. This might be due to the small number of samples, and other vertebrates may be the definitive host for *D. hongkongensis*. Interestingly, many filarial nematodes contain *Wolbachia*, an obligate bacterial endosymbiont, which plays an important role in the biological development of filarial nematodes and is involved in targeted therapy against them, leading to the loss of worm fertility and viability upon antibiotic treatment [23,24]. *Wolbachia* can be classified into at least seven supergroups based on the *FtsZ* and *Wolbachia* surface protein (*wsp*) sequences [46,47,48,49]. *Wolbachia* supergroups C and D have been detected in nematodes, whereas four supergroups (i.e., A, B, E, and H) have only been found in arthropods [50]. Furthermore, supergroup F has been found in both arthropod and nematode species [47]. Our results showed the preliminary study of the phylogenetic tree of all positive *Wolbachia* from 33 (57.89%), 11 (19.30%), and 3 (5.26%) samples of *D. immitis, B. pahangi*, and *B. malayi,* respectively. These were classified into a clade of supergroup C, which is closely related to the *Wolbachia* endosymbiont of *D. immitis* from Italy (accession no. AJ495000) and the *Wolbachia* endosymbiont of *Onchocerca ochengi* (accession no. HE660029) and *Onchocerca volvulus* (accession no. HG810405) from the United Kingdom [51]. In terms of *Wolbachia* and nematode symbiosis, it was revealed that *Wolbachia* are obligate mutualists for filariae worms and are necessary for the development, fertility, and vitality of adult filariae [52]. *Wolbachia* are found in all developmental stages of the filarial nematode but rapidly increase in number as the nematode transitions from its insect vectors to mammalian hosts. The *Wolbachia* titres further increase during the development of the larvae into the adult stages. The high titre of *Wolbachia* is also found within oocytes and infected embryos [53,54]. This study highlights PCR as the diagnostic tool for filariasis and the presence of *Wolbachia* in dogs from the Bangkok Metropolitan Region, Thailand. Regarding the limitations of the study, the examination of additional dog blood samples from geographical regions of Thailand is required for a more complete and robust understanding of the phylogeography of *Wolbachia* and filariasis in Thailand. The use of bacteria as a new control trend simultaneously targeting the vector and filarial parasites should also be explored in the future.

## 4. Materials and Methods

### 4.1. Ethics Statement

The study was approved by the animal research ethics committee of Chulalongkorn University Animal Care and Use Protocol (CU-ACUP), Faculty of Medicine, Chulalongkorn University, Bangkok, Thailand (COA No. 022/2561).

### 4.2. Sample Collection

A total of 57 blood samples were collected from domestic dogs in the Bangkok Metropolitan Region, Thailand by collaborating with veterinarians at the Small Animal Teaching Hospital, Chulalongkorn University and Faculty of Veterinary Science, Mahidol University Salaya Campus. The specimens were stored at 4 °C and transferred to the laboratory of the Vector Biology and Vector Borne Disease Research Unit, Department of Parasitology, Faculty of Medicine, Chulalongkorn University for filarial worm detection (Table S1). These samples were differentiated for morphological identification and molecular detection.

### 4.3. Microscopic Examination 

Drops of blood were smeared on glass slides. Thick smears were fixed with absolute methanol (Sigma-Aldrich, St. Louis, MO, USA) for one minute, stained with Giemsa (Sigma-Aldrich, St. Louis, MO, USA) in a phosphate buffer (with a pH of 7.2), examined under a light microscope (CH20, Olympus, Tokyo, Japan), and morphologically identified for microfilariae with or without sheath species using the taxonomic key [55,56,57].

### 4.4. DNA Extraction

DNA was extracted from the dog blood samples by using a blood DNA extraction kit (Invisorb Spin Blood Mini Kit, STRATEC Molecular, Berlin, Germany) according to the manufacturer’s instructions. Extracted DNA was eluted in 50 μL of elution buffer. Extracted DNA samples were kept at −80 °C for long-term storage.

### 4.5. Molecular Identification of Canine Filarial 

The microfilariae DNA samples were amplified for partial *COI* genes and *ITS1* regions. For the *COI* gene, two oligonucleotide primers from previous studies [58], COI-int-F (5′-TGATTGGTGGTTT TGGTAA-3′) and COI-int-R (5′-ATAAGTACGAGTATCAATATC-3′), were used for PCR amplification. The PCR reactions were performed in a total volume of 20 μL containing 50 ng of DNA template, 10× buffer (Thermo Fisher Scientific, Waltham, MA, USA), 25 mM of MgCl_2_ (Thermo Fisher Scientific, Waltham, MA, USA), 2 mM of dNTPs (GeneAll, Korea), 10 μM each of forward and reverse primers, and five units of *Taq* DNA polymerase (Thermo Fisher Scientific, Waltham, MA, USA). The thermal profile was as follows: 94 °C for 3 min; 40 cycles of 94 °C for 45 s, 52 °C for 45 s, and 72 °C for 90 s; and 72 °C for 7 min. The *ITS1* region was PCR-amplified using forward primer ITS1-F (5′-GGTGAACCTGCGGAAGGATC-3′) and reverse primer ITS1-R (5′-CTCAATGCGTCTGCAATTCGC -3′), and this was performed in 25 μL of reaction mixture containing 50 ng of DNA template, 10× buffer (Thermo Fisher Scientific, Waltham, MA, USA), 25 mM of MgCl_2_ (Thermo Fisher Scientific, MA, USA), 2 mM of dNTPs (GeneAll, Korea), 10 μM of each primer, and five units of *Taq* DNA polymerase (Thermo Fisher Scientific, Waltham, MA, USA). The PCR conditions were 94 °C for 5 min; 35 cycles of 94 °C for 30 s, 58 °C for 30 s, and 72 °C for 45 s; and 72 °C for 10 min [31]. The positive controls used in this study consisted of *D. immitis*, *B. pahangi*, and *B. malayi* DNA, whereas double-distilled water was used as a negative control. The PCR products were analyzed via 1.5% agarose gel electrophoresis, stained with ethidium bromide, and visualized with Quantity One Quantification Analysis Software Version 4.5.2 (Gel DocEQ System; Bio-Rad, Hercules, CA, USA). 

### 4.6. Wolbachia Bacterial Detection

The nested PCR screening for *Wolbachia* bacteria was amplified by using two pairs of primer. Two primers, including Wol1-fwd (5′-CCTGTACTATATCCAAGAATTACTG-3′) and Wol1-R (5′-AC TATCCTTTATATGTTCCATAATTTC-3), were used for the first round. For the second round of amplification, two primers, Wol7-fwd (5′-GGTGGAAATGCTGTGAATAAC-3′) and Wol7-R (5′-AGC ACCGAGCCCTTTAG-3′), that were previously designed on the *FtsZ* region were used [59]. The PCR mixtures were 25 μL in total, consisting of 50 ng of DNA template, 10× buffer (Thermo Fisher Scientific, Waltham, MA, USA), 25 mM of MgCl_2_ (Thermo Fisher Scientific, Waltham, MA, USA), 2 mM of dNTPs (GeneAll, Korea), 300 nM of each primer, and five units of *Taq* DNA Polymerase (Thermo Fisher Scientific, Waltham, MA, USA). The thermal profiles used were as described previously. The positive control for *Wolbachia* PCR used was the DNA of *Wolbachia* supergroup B in *Aedes albopictus* mosquitoes, while double-distilled water was used as a negative control. The PCR products were examined using 1.5% agarose gel electrophoresis, stained with ethidium bromide, and visualized under ultraviolet transilluminator Quantity One Quantification Analysis Software Version 4.5.2 (Gel DocEQ System; Bio-Rad, Hercules, CA, USA).

### 4.7. Cloning and Sequencing

All positive PCR amplicons were ligated into pGEM-T Easy Vector (Promega, Madison, WI, USA), and the recombinant plasmids were used to transform a competent *Escherichia coli* DH5α strain. Transformed cells were cultured and recombinant plasmids were then extracted using Invisorb^®^ Spin Plasmid Mini kit (STRATEC Molecular, Berlin, Germany) following the manufacturer’s instructions. Plasmids were sequenced by a commercial service at AITBIOTECH, Singapore and MACROGEN, Korea.

### 4.8. Sequence Analysis and Phylogenetic Tree Construction

The sequences were aligned using BioEdit Sequence Alignment Editor Version 7.2.5 [60]. The phylogenetic trees were constructed using the maximum-likelihood method with IQ-TREE on the IQ-TREE web server (http://iqtree.cibiv.univie.ac.at/) with 1000 ultrafast bootstrap replicates. The best-fit model of substitution was found using the auto function on the IQ-TREE web server [61]. The phylogenetic tree was finally viewed and edited with FigTree v1.4.4 software (http://tree.bio.ed.ac.uk/software/figtree/). 

## 5. Conclusions

Between the aforementioned tested PCR methods, the *COI*-based method was more suitable for diagnosing canine filaria than the *ITS1*-based method. This *COI*-PCR method could differentiate *D. immitis, B. malayi*, and *B. pahangi* by their amplicon sizes in a single-tube PCR. This method could be useful for the epidemiological survey of filarial infection in humans, mosquitoes, and other reservoirs in Thailand. In addition, the detection of *Wolbachia* within filaria-infected dogs in our locality shows the potential of using the bacteria as a new control trend to be done simultaneously with targeting the vector and filarial parasites.

## Figures and Tables

**Table 1:** The results of microscopic examinations and PCR for the detection of filarial worm DNA from blood dog specimens (n = 57). Positive controls used the DNA of *Dirofilaria immitis, Brugia pahangi,* and *Brugia malayi.*

Species	Microscopic Examination (%)	Molecular Detection (%)	
		*COI*	*ITS1*
*Dirofilaria* spp.	11(19.30%)		
*Brugia* spp.	5 (8.77%)		
*D. immitis*		33 (57.89%)	30 (52.63%)
*B. pahangi*		13 (22.81%)	11 (19.30%)
*B. malayi*		4 (7.02%)	0
Total	16 (28.07%)	50 (87.72%)	41 (71.93%)

**Table 2:** Filarial worm tested for infection with *Wolbachia* using nested PCR of the *FtsZ* gene (n = 57). Positive controls used the DNA of *Wolbachia* supergroup B in *Aedes albopictus* mosquitoes.

Species	Positive for *Wolbachia* (%)
*D. immitis*	33 (57.89%)
*B. pahangi*	11 (19.30%)
*B. malayi*	3 (5.26%)
Total	47 (82.45%)

## Supplementary Materials

The following are available online at https://www.mdpi.com/2076-0817/8/3/114/s1, Figure S1: PCR amplification of the partial *COI* gene for filarial nematode, Figure S2: PCR amplification of the *ITS1* region for the filarial nematode, Figure S3: Sequence alignment of the filaria nematode based on the partial *COI* gene, Figure S4: Sequence alignment of the filaria nematode based on the *ITS1* region, Figure S5: PCR amplicons of the *FtsZ* specific to *Wolbachia*, Figure S6: Sequence alignment of *Wolbachia* bacteria based on the *FtsZ* gene, Table S1: Raw data of samples.

## References

[1] World Health Organization Lymphatic Filariasis. https://www.who.int/news-room/fact-sheets/detail/lymphatic-filariasis.

[2] Kaikuntod M., Thongkorn K., Tiwananthagorn S., Boonyapakorn C. (2018). Filarial worms in dogs in Southeast Asia. Vet. Integr. Sci..

[3] Mak J.W., Yen P.K., Lim K.C., Ramiah N. (1980). Zoonotic implications of cats and dogs in filarial transmission in Peninsular Malaysia. Trop. Geogr. Med..

[4] Irwin P.J. (2002). Companion animal parasitology: A clinical perspective. Int. J. Parasitol. Res..

[5] Kelly J.D. (1979). Canine heart worm disease. Current Veterinary Therapy VII.

[6] Rishniw M., Barr S.C., Simpson K.W., Frongillo M.F., Franz M., Dominguez Alpizar J.L. (2006). Discrimination between six species of canine microfilariae by a single polymerase chain reaction. Vet. Parasitol..

[7] Kanjanopas K., Choochote W., Jitpakdi A., Suvannadabba S., Loymak S., Chungpivat S., Nithiuthai S. (2001). *Brugia malayi* in a naturally infected cat from Narathiwat province, southern Thailand. Southeast. Asian J. Trop. Med. Public Health.

[8] Kamyingkird K., Junsiri W., Chimnoi W., Kengradomkij C., Saengow S., Sangchuto K., Ka jeerum W., Pangjai D., Nimsuphan B., Inpankeaw T. (2017). Prevalence and risk factors associated with *Dirofilaria immitis* infection in dogs and cats in Songkhla and Satun provinces, Thailand. Agric. Nat. Resour..

[9] Wongkamchai S., Nochotea H., Foongladda S., Dekumyoy P., Thammapalo S., Boitanoa J.J., Choochotee W. (2014). A high resolution melting real time PCR for mapping of filaria infection in domestic cats living in brugian filariosis-endemic areas. Vet. Parasitol..

[10] Simón F., Morchón R., González-Miguel J., Marcos-Atxutegi C., Siles-Lucas M. (2009). What is new about animal and human dirofilariosis?. Trends. Parasitol..

[11] Ichimori K., King J.D., Engels D., Yajima A., Mikhailov A., Lammie P., Ottesen E.A. (2014). Global programme to eliminate lymphatic filariasis: The processes underlying programme success. PLoS. Negl. Trop. Dis..

[12] World Health Organization (2017). Essential Medicines Donated to Control, Eliminate and Eradicate Neglected Tropical Diseases.

[13] World Health Organization (2013). Lymphatic Filariasis: A Handbook of Practical Entomology for National Lymphatic Filariasis Elimination Programmes.

[14] Famakinde D.O. (2018). Mosquitoes and the Lymphatic Filarial Parasites: Research Trends and Budding Roadmaps to Future Disease Eradication. Trop. Med. Infect. Dis..

[15] Pradatsundarasar A. (1955). *Dirofilaria* infection in man: Report of a case. J. Med. Assoc. Thailand.

[16] Jariya P., Sucharit S. (1983). *Dirofilaria repens* from the eyelid of a woman in Thailand. Am. J. Trop. Med. Hyg..

[17] Sukudom P., Phumee A., Siriyasatien P. (2018). First report on subconjunctival dirofilariasis in Thailand caused by a *Dirofilaria* sp. closely related to *D*. *hongkongensis*. Acad. J. Sci. Res..

[18] Taylor M.J., Bandi C., Hoerauf A. (2005). *Wolbachia* bacterial endosymbionts of filarial nematodes. Adv. Parasitol..

[19] Taylor M.J., Voronin D., Johnston K.L., Ford L. (2013). *Wolbachia* filarial interactions. Cell. Microbiol..

[20] Bandi C., Trees A.J., Brattig N.W. (2001). *Wolbachia* in filarial nematodes: Evolutionary aspects and implications for the pathogenesis and treatment of filarial diseases. Vet. Parasitol..

[21] Werren J.H., Baldo L., Clark M.E. (2008). *Wolbachia*: Master manipulators of invertebrate biology. Nat. Rev. Microbiol..

[22] Clark E.L., Karley A.J., Hubbard S.F. (2010). Insect endosymbionts: Manipulators of insect herbivore trophic interactions?. Protoplasma.

[23] Slatko B.E., Taylor M.J., Foster J.M. (2010). The *Wolbachia* endosymbiont as an anti-filarial nematode target. Symbiosis.

[24] Pfarr K.M., Hoerauf A.M. (2006). Antibiotics which target the *Wolbachia* endosymbionts of filarial parasites: A new strategy for control of filariasis and amelioration of pathology. Mini. Rev. Med. Chem..

[25] Ravindran R., Varghese S., Nair S.N., Balan V.M., Lakshmanan B., Ashruf R.M., Kumar S.S., Gopalan A.K., Nair A.S., Malayil A. (2014). Canine filarial infections in a human *Brugia malayi* endemic area of India. Biomed. Res. Int..

[26] Megat Abd Rani P.A., Irwin P.J., Gatne M., Coleman G.T., McInnes L.M., Traub R.J. (2010). A survey of canine filarial diseases of veterinary and public health significance in India. Parasit. Vectors.

[27] Saseendranath M.R., Varghese C.G., Jayakumar K.M. (1986). Incidence of canine dirofilariosis in Trichur, Kerala. Indian J. Vet. Med..

[28] Otranto D., Dantas-Torres F., Breitschwerdt E.B. (2009). Managing canine vector-borne diseases of zoonotic concern: Part one. Trends Parasitol..

[29] Rosenblatt J.E. (2009). Laboratory diagnosis of infections due to blood and tissue parasites. Clin. Infect. Dis..

[30] Ricciardi A., Ndao M. (2015). Diagnosis of Parasitic Infections: What’s Going On?. J. Biomol. Screen..

[31] Nuchprayoon S., Junpee A., Poovorawan Y., Scott A.L. (2005). Detection and differentiation of filarial parasites by universal primers and polymerase chain reaction-restriction fragment length polymorphism analysis. Am. J. Trop. Med. Hyg..

[32] Magnis J., Lorentz S., Guardone L., Grimm F., Magi M., Naucke T.J., Deplazes P. (2013). Morphometric analyses of canine blood microfilariae isolated by the Knott’s test enables *Dirofilaria immitis* and *D*. *repens* species-specific and Acanthocheilonema (syn. Dipetalonema) genus-specific diagnosis. Parasit. Vectors.

[33] Hoch H., Strickland K. (2008). Canine and feline dirofilariasis: Life cycle, pathophysiology, and diagnosis. Compend. Contin. Educ. Vet..

[34] Casiraghi M., Bazzocchi C., Mortarino M., Ottina E., Genchi C. (2006). A simple molecular method for discriminating common filarial nematodes of dogs (*Canis familiaris*). Vet. Parasitol..

[35] Wang S., Zhang N., Zhang Z., Wang D., Yao Z., Zhang H., Ma J., Zheng B., Ren H., Liu S. (2016). Prevalence of *Dirofilaria immitis* infection in dogs in Henan province, central China. Parasite.

[36] Byeon K.H., Kim B.J., Kim S.M., Yu H.S., Jeong H.J., Ock M.S. (2007). A serological survey of *Dirofilaria immitis* infection in pet dogs of Busan, Korea, and effects of chemoprophylaxis. Korean J. Parasitol..

[37] Khedri J., Radfar M.H., Borji H., Azizzadeh M., Akhtardanesh B. (2014). Canine Heartworm in Southeastern of Iran with Review of disease distribution. Iran J. Parasitol..

[38] Alho A.M., Landum M., Ferreira C., Meireles J., Goncalves L., de Carvalho L.M., Belo S. (2014). Prevalence and seasonal variations of canine dirofilariosis in Portugal. Vet. Parasitol..

[39] Bacsadi A., Papp A., Szeredi L., Toth G., Nemes C., Imre V., Tolnai Z., Szell Z., Sreter T. (2016). Retrospective study on the distribution of *Dirofilaria immitis* in dogs in Hungary. Vet. Parasitol..

[40] Tiawsirisup S., Thanapaisarnkit T., Varatorn E., Apichonpongsa T., Bumpenkiattikun N., Rattanapuchpong S., Chungpiwat S., Sanprasert V., Nuchprayoon S. (2015). Canine Heartworm (*Dirofilaria immitis*) Infection and Immunoglobulin G Antibodies Against *Wolbachia* (Rickettsiales: Rickettsiaceae) in Stray Dogs in Bangkok, Thailand. Thai. J. Vet. Med..

[41] Ambily V.R., Pillai U.N., Arun R., Pramod S., Jayakumar K.M. (2011). Detection of human filarial parasite *Brugia malayi* in dogs by histochemical staining and molecular techniques. Vet. Parasitol..

[42] Chungpivat S., Taweethavonsawat P. (2008). The differentiation of microfilariae in dogs and cats using Giemsa’s staining and the detection of acid phosphatase activity. J. Thai Vet. Pract..

[43] Thanchomnang T., Intapan P.M., Chungpivat S., Lulitanond V., Maleewong W. (2010). Differential detection of *Brugia malayi* and *Brugia pahangi* by real-time fluorescence resonance energy transfer PCR and its evaluation for diagnosis of *B*. *pahangi*-infected dogs. Parasitol. Res..

[44] Chansiri K., Tejangkura T., Kwaosak P., Sarataphan N., Phantana S., Sukhumsirichart W. (2002). PCR based method for identification of zoonostic *Brugia malayi* microfilariae in domestic cats. Mol. Cell. Probes.

[45] Oh I.Y., Kim K.T., Sung H.J. (2017). Molecular Detection of *Dirofilaria immitis* Specific Gene from Infected Dog Blood Sample Using Polymerase Chain Reaction. Iran. J. Parasitol..

[46] Lo N., Casiraghi M., Salati E., Bazzocchi C., Bandi C. (2002). How many *Wolbachia* supergroups exist?. Mol. Biol Evol..

[47] Casiraghi M., Bordenstein S.R., Baldo L., Lo N., Beninati T., Wernegreen J.J., Werren J.H., Bandi C. (2005). Phylogeny of *Wolbachia pipientis* based on gltA, groEL and ftsZ gene sequences: Clustering of arthropod and nematode symbionts in the F supergroup, and evidence for further diversity in the *Wolbachia* tree. Microbiology.

[48] Bordenstein S.R., Paraskevopoulos C., Hotopp J.C., Sapountzis P., Lo N., Bandi C., Tettelin H., Werren J.H., Bourtzis K. (2009). Parasitism and mutualism in *Wolbachia*: What the phylogenomic trees can and cannot say. Mol. Biol. Evol..

[49] Baldo L., Werren J.H. (2007). Revisiting *Wolbachia* supergroup typing based on WSP: Spurious lineages and discordance with MLST. Curr. Microbiol..

[50] Bandi C., Anderson T.J., Genchi C., Blaxter M.L. (1998). Phylogeny of *Wolbachia* in filarial nematodes. Proc. Biol. Sci..

[51] Darby A.C., Armstrong S.D., Bah G.S., Kaur G., Hughes M.A., Kay S.M., Koldkjær P., Rainbow L., Radford A.D., Blaxter M.L. (2012). Analysis of gene expression from the *Wolbachia* genome of a filarial nematode supports both metabolic and defensive roles within the symbiosis. Genome Res..

[52] Lefoulon E., Bain O., Makepeace B.L., d’Haese C., Uni S., Martin C., Gavotte L. (2016). Breakdown of coevolution between symbiotic bacteria *Wolbachia* and their filarial hosts. PeerJ.

[53] Fenn K., Blaxter M. (2004). Quantification of *Wolbachia* bacteria in *Brugia malayi* through the nematode lifecycle. Mol. Biochem. Parasitol..

[54] McGarry H.F., Egerton G.L., Taylor M.J. (2004). Population dynamics of *Wolbachia* bacterial endosymbionts in *Brugia malayi*. Mol. Biochem. Parasitol..

[55] Nelson G.S. (1959). The identification of infective filarial larvae in mosquitoes: With a note on the species found in “wild” mosquitoes on the Kenya coast. J. Helmintho..

[56] Yen P.K.F., Zaman V., Mak J.W. (1982). Identification of some common infective filarial larvae in Malaysia. J. Helminthol..

[57] Orihel T.C., Ash L.R., Ramachandran C.P., Ottesen E.A. (1997). Bench Aids for the Diagnosis of Filarial Infections.

[58] Casiraghi M., Anderson T.J., Bandi C., Bazzocchi C., Genchi C. (2001). A phylogenetic analysis of filarial nematodes: Comparison with the phylogeny of *Wolbachia* endosymbionts. Parasitology.

[59] Turba M.E., Zambon E., Zannoni A., Russo S., Gentilini F. (2012). Detection of *Wolbachia* DNA in blood for diagnosing filaria-associated syndromes in cats. J. Clin. Microbiol..

[60] Hall T.A. (1999). BioEdit: A user-friendly biological sequence alignment editor and analysis program for Windows 95/98/NT. Nucleic. Acids. Symp. Ser..

[61] Trifinopoulos J., Nguyen L.T., von Haeseler A., Minh B.Q. (2016). W-IQ-TREE: A fast online phylogenetic tool for maximum likelihood analysis. Nucleic. Acids. Res..

