# Supplementary material for: Molecular Analysis of Canine Filaria and Its Wolbachia Endosymbionts in Domestic Dogs Collected from Two Animal University Hospitals in Bangkok Metropolitan Region, Thailand
Source: Pathogens. 2019 Jul 29;8(3):114. doi: 10.3390/pathogens8030114 (PMC6789508; doi:10.3390/pathogens8030114)
Supplement: Supplementary file 1 [file pathogens-08-00114-s001.zip › Figure supplement/Supplementary Materials.docx]

**Supplementary Materials:**


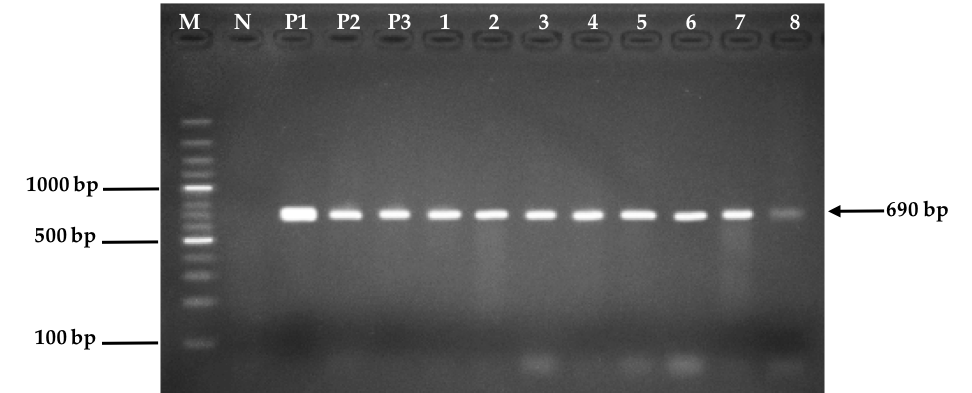


**Figure S1:** PCR amplification of the partial *COI* gene for filarial nematode. Lane M: Molecular mass marker (100 base pairs [bp]). Lane P1: *D. immitis* positive control. Lane P2: *B. pahangi* positive control. Lane P3: *B. malayi* positive control. Lane N: Negative control. Lanes 1–8: Positive samples.


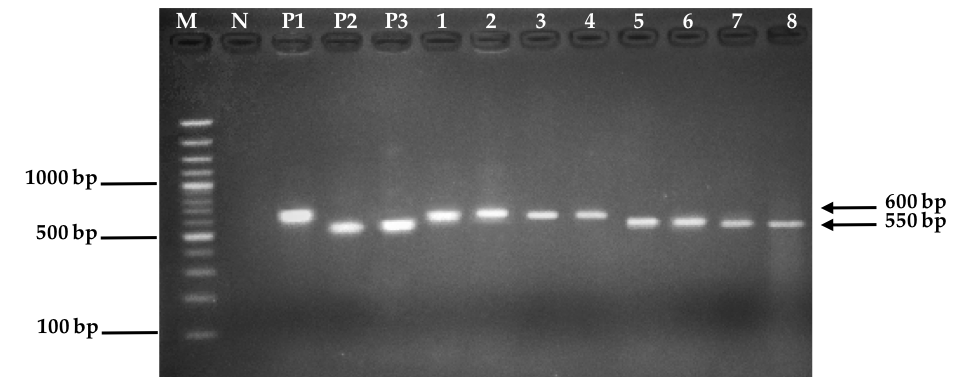


**Figure S2:** PCR amplification of the *ITS1* region for the filarial nematode. Lane M: Molecular mass marker (100 base pairs [bp]). Lane P1: *D. immitis* positive control. Lane P2: *B. pahangi* positive control. Lane P3: *B. malayi* positive control. Lane N: Negative control. Lanes 1–8: Positive samples.


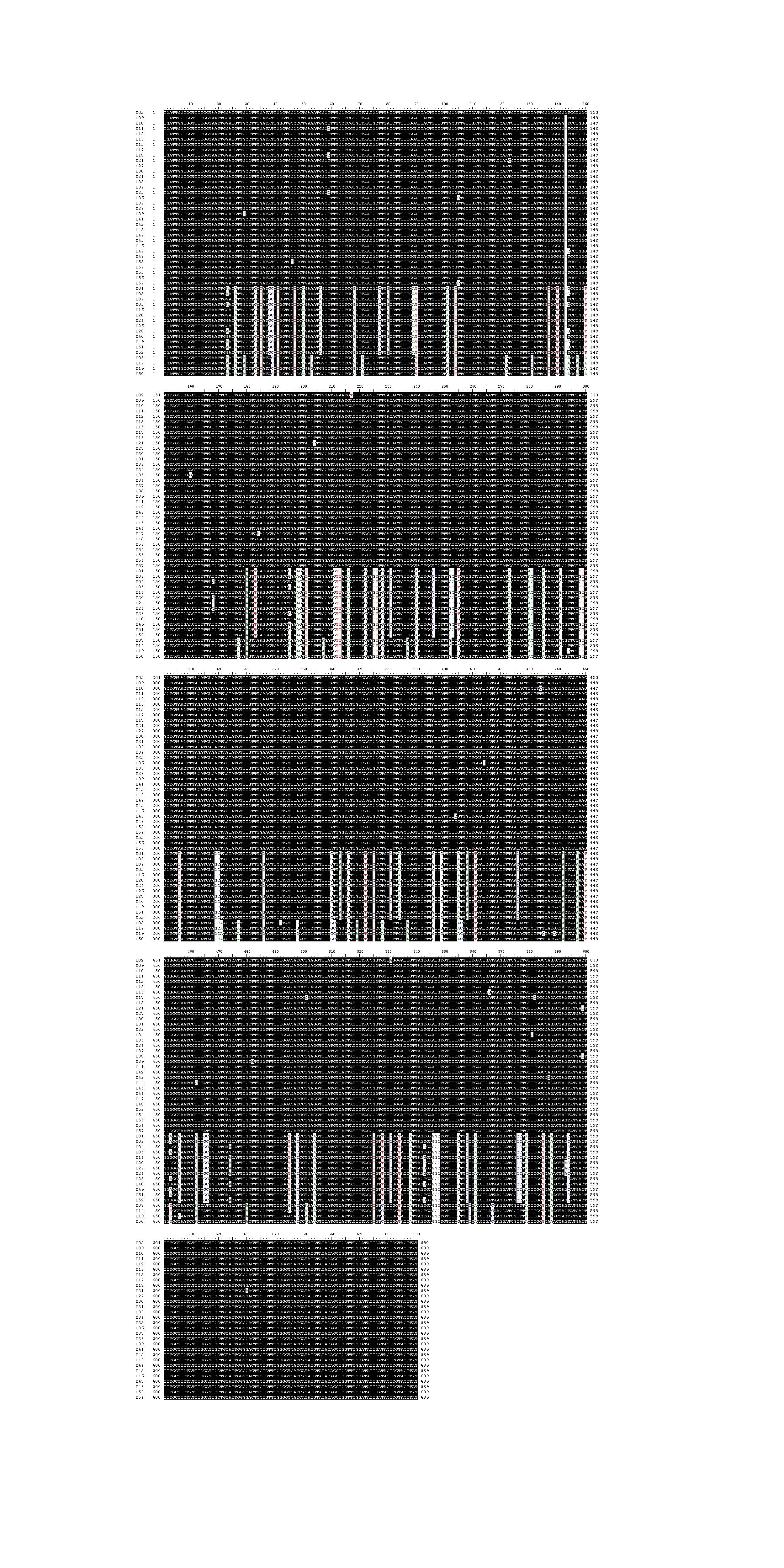


**Figure S3:** Sequence alignment of the filaria nematode based on the partial *COI* gene.


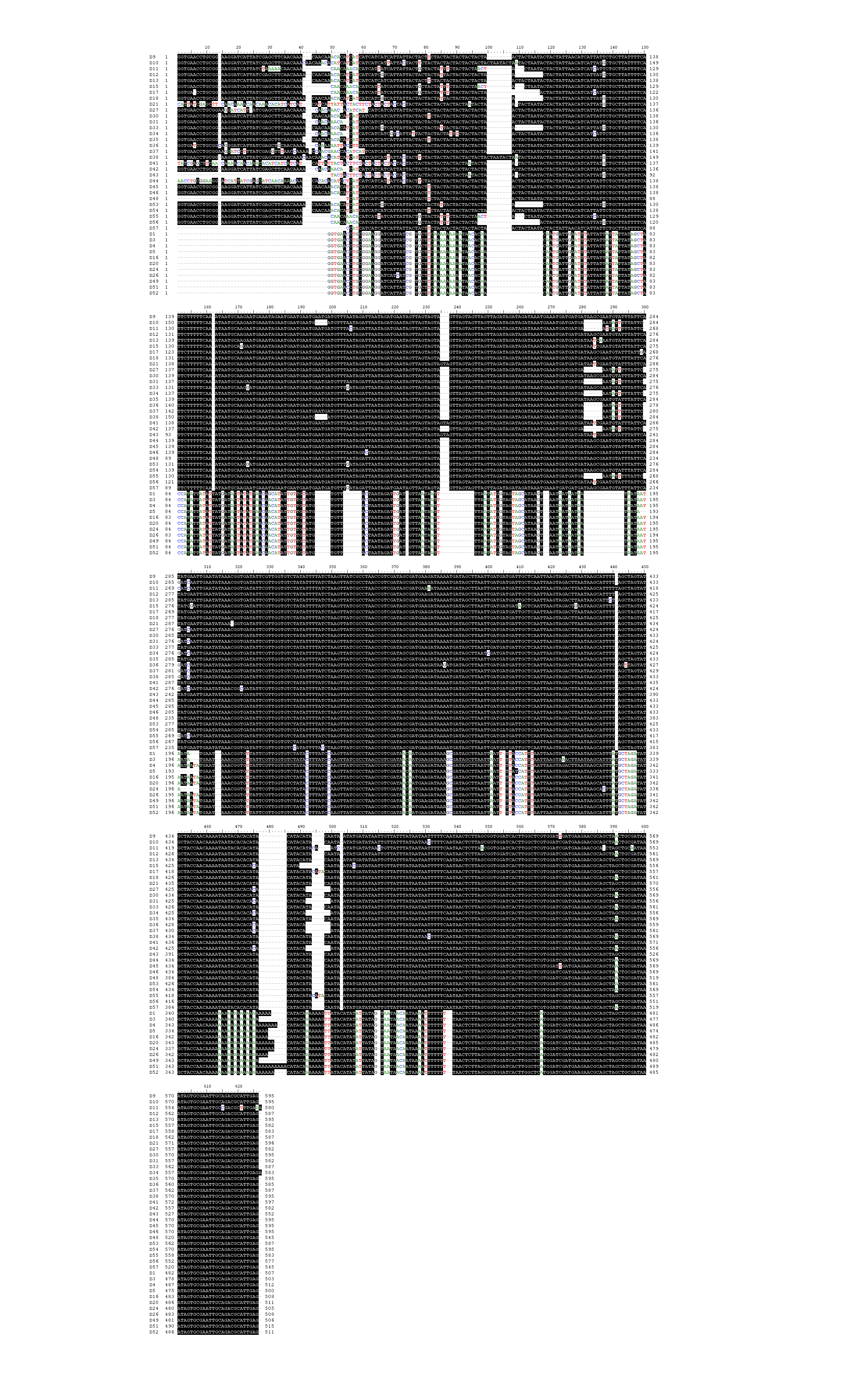


**Figure S4:** Sequence alignment of the filaria nematode based on the *ITS1* region


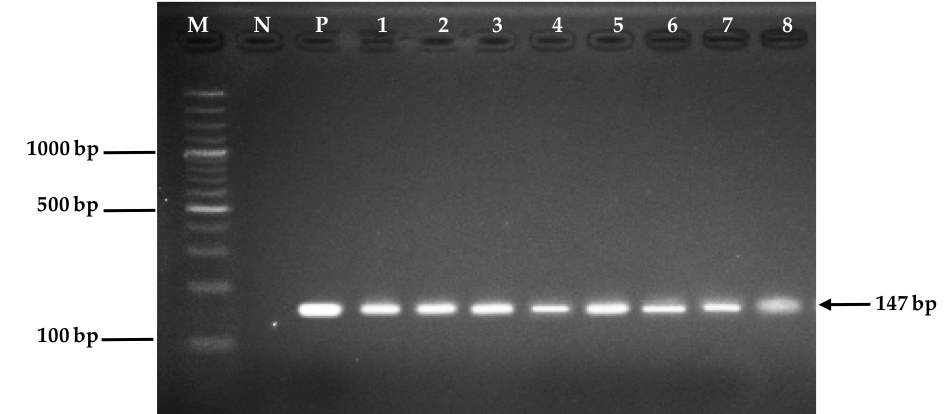


**Figure S5:** PCR amplicons of the *FtsZ* specific to *Wolbachia*. Lane M: molecular mass marker (100 base pairs [bp]). Lane P1: *Wolbachia* positive control. Lane N: Negative control. Lanes 1–8: Positive samples.


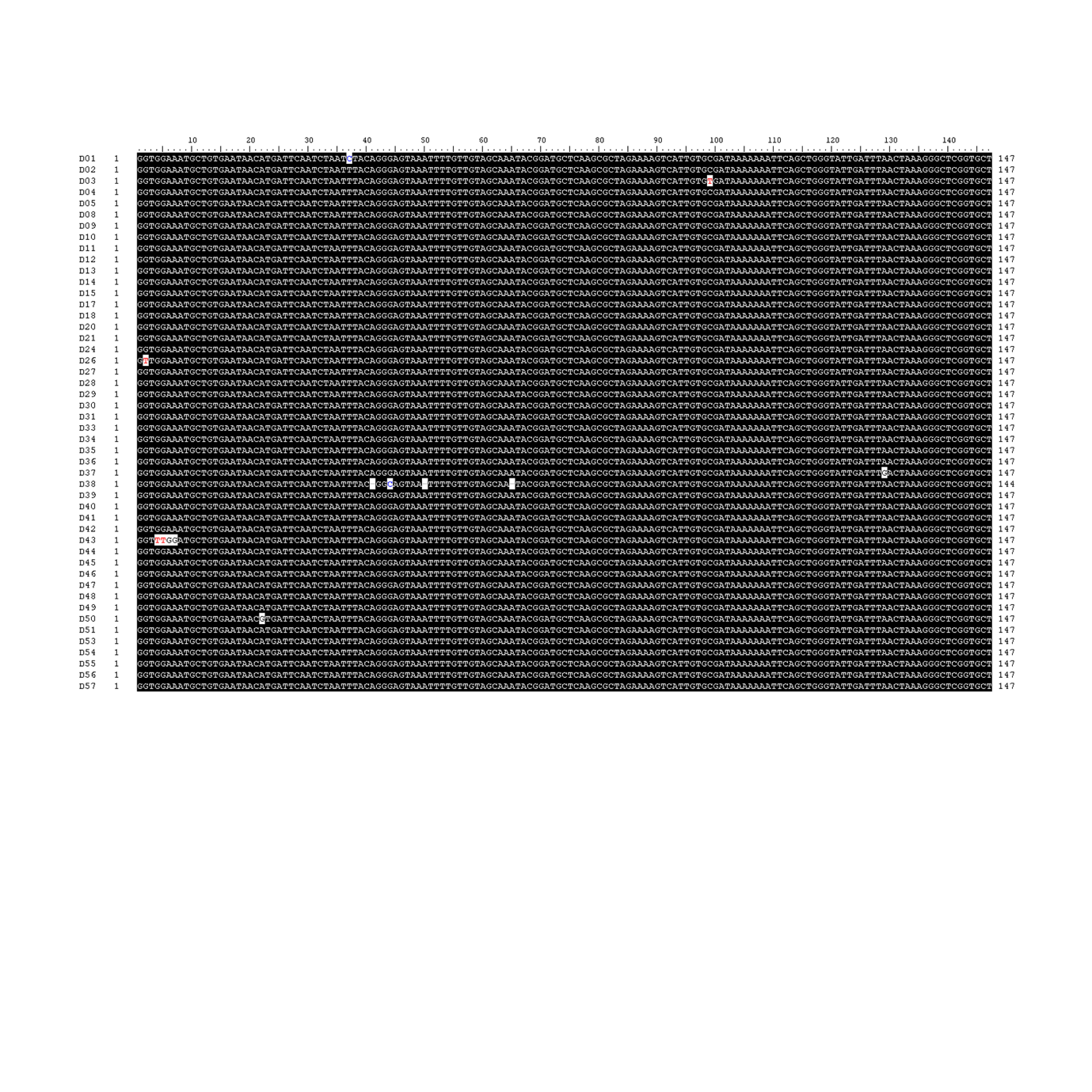


**Figure S6:** Sequence alignment of *Wolbachia* bacteria based on the *FtsZ* gene.

**Table S1:** Raw data of samples.

| **CODE** | **COI gene** | **ITS1 region** | **FtsZ gene** |
| --- | --- | --- | --- |
|  |  |  |  |
| D01 | *B. pahangi* | *B. pahangi* | *Wolbachia* |
| D02 | *D. immitis* | - | *Wolbachia* |
| D03 | *B. pahangi* | *B. pahangi* | *Wolbachia* |
| D04 | *B. pahangi* | *B. pahangi* | *Wolbachia* |
| D05 | *B. pahangi* | *B. pahangi* | *Wolbachia* |
| D06 | *B. malayi* | **-** | *Wolbachia* |
| D07 | **-** | **-** | **-** |
| D08 | **-** | **-** | **-** |
| D09 | *D. immitis* | *D. immitis* | *Wolbachia* |
| D10 | *D. immitis* | *D. immitis* | *Wolbachia* |
| D11 | *D. immitis* | *D. immitis* | *Wolbachia* |
| D12 | *D. immitis* | *D. immitis* | *Wolbachia* |
| D13 | *D. immitis* | *D. immitis* | *Wolbachia* |
| D14 | *B. malayi* | - | *Wolbachia* |
| D15 | *D. immitis* | *D. immitis* | *Wolbachia* |
| D16 | *B. pahangi* | *B. pahangi* | **-** |
| D17 | *D. immitis* | *D. immitis* | *Wolbachia* |
| D18 | *D. immitis* | *D. immitis* | *Wolbachia* |
| D19 | **-** | **-** | **-** |
| D20 | *B. pahangi* | *B. pahangi* | *Wolbachia* |
| D21 | *D. immitis* | *D. immitis* | *Wolbachia* |
| D22 | **-** | **-** | **-** |
| D23 | **-** | **-** | **-** |
| D24 | *B. pahangi* | *B. pahangi* | *Wolbachia* |
| D25 | - | - | **-** |
| D26 | *B. pahangi* | *B. pahangi* | *Wolbachia* |
| D27 | *D. immitis* | *D. immitis* | *Wolbachia* |
| D28 | *B. pahangi* | - | *Wolbachia* |
| D29 | *B. malayi* | *B. pahangi* | *Wolbachia* |
| D30 | *D. immitis* | *D. immitis* | *Wolbachia* |
| D31 | *D. immitis* | *D. immitis* | *Wolbachia* |
| D32 | - | - | **-** |
| D33 | *D. immitis* | *D. immitis* | *Wolbachia* |
| D34 | *D. immitis* | *D. immitis* | *Wolbachia* |
| D35 | *D. immitis* | *D. immitis* | *Wolbachia* |
| D36 | *D. immitis* | *D. immitis* | *Wolbachia* |
| D37 | *D. immitis* | *D. immitis* | *Wolbachia* |
| D38 | *D. immitis* | *D. immitis* | *Wolbachia* |
| D39 | *D. immitis* | - | *Wolbachia* |
| D40 | *B. pahangi* | - | *Wolbachia* |
| D41 | *D. immitis* | *D. immitis* | *Wolbachia* |
| D42 | *D. immitis* | *D. immitis* | *Wolbachia* |
| D43 | *D. immitis* | *D. immitis* | *Wolbachia* |
| D44 | *D. immitis* | *D. immitis* | *Wolbachia* |
| D45 | *D. immitis* | *D. immitis* | *Wolbachia* |
| D46 | *D. immitis* | *D. immitis* | *Wolbachia* |
| D47 | *D. immitis* | - | *Wolbachia* |
| D48 | *D. immitis* | *D. immitis* | *Wolbachia* |
| D49 | *B. pahangi* | *B. pahangi* | *Wolbachia* |
| D50 | *B. malayi* | - | *Wolbachia* |
| D51 | *B. pahangi* | *B. pahangi* | *Wolbachia* |
| D52 | *B. pahangi* | *B. pahangi* | **-** |
| D53 | *D. immitis* | *D. immitis* | *Wolbachia* |
| D54 | *D. immitis* | *D. immitis* | *Wolbachia* |
| D55 | *D. immitis* | *D. immitis* | *Wolbachia* |
| D56 | *D. immitis* | *D. immitis* | *Wolbachia* |
| D57 | *D. immitis* | *D. immitis* | *Wolbachia* |
