# Supplementary material for: Molecular Analysis of Canine Filaria and Its Wolbachia Endosymbionts in Domestic Dogs Collected from Two Animal University Hospitals in Bangkok Metropolitan Region, Thailand
Source: Pathogens. 2019 Jul 29;8(3):114. doi: 10.3390/pathogens8030114 (PMC6789508; doi:10.3390/pathogens8030114)
Supplement: Supplementary file 1 [file pathogens-08-00114-s001.zip › Figure supplement/Supplementary Materials.pdf]

Supplementary Materials:

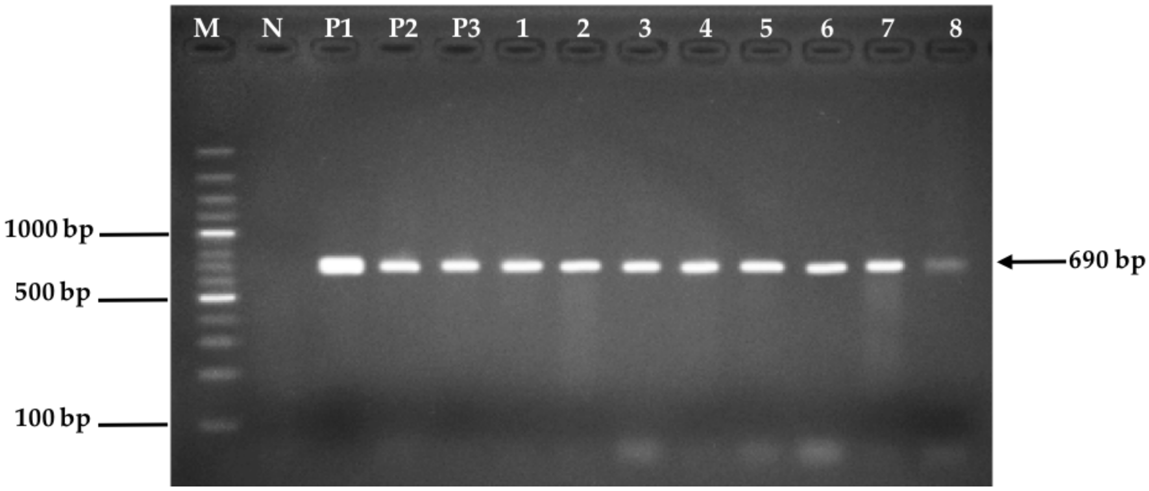

Figure S1: PCR amplification of the partial *COI* gene for filarial nematode. Lane M: Molecular mass marker (100 base pairs [bp]). Lane P1: *D. immitis* positive control. Lane P2: *B. pahangi* positive control. Lane P3: *B. malayi* positive control. Lane N: Negative control. Lanes 1–8: Positive samples.

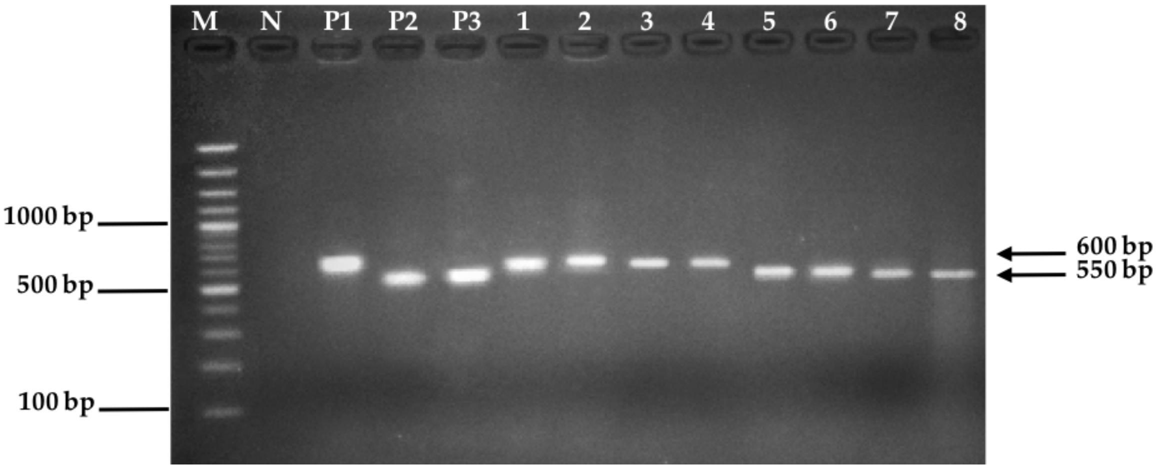

Figure S2: PCR amplification of the *ITS1* region for the filarial nematode. Lane M: Molecular mass marker (100 base pairs [bp]). Lane P1: *D. immitis* positive control. Lane P2: *B. pahangi* positive control. Lane P3: *B. malayi* positive control. Lane N: Negative control. Lanes 1–8: Positive samples.

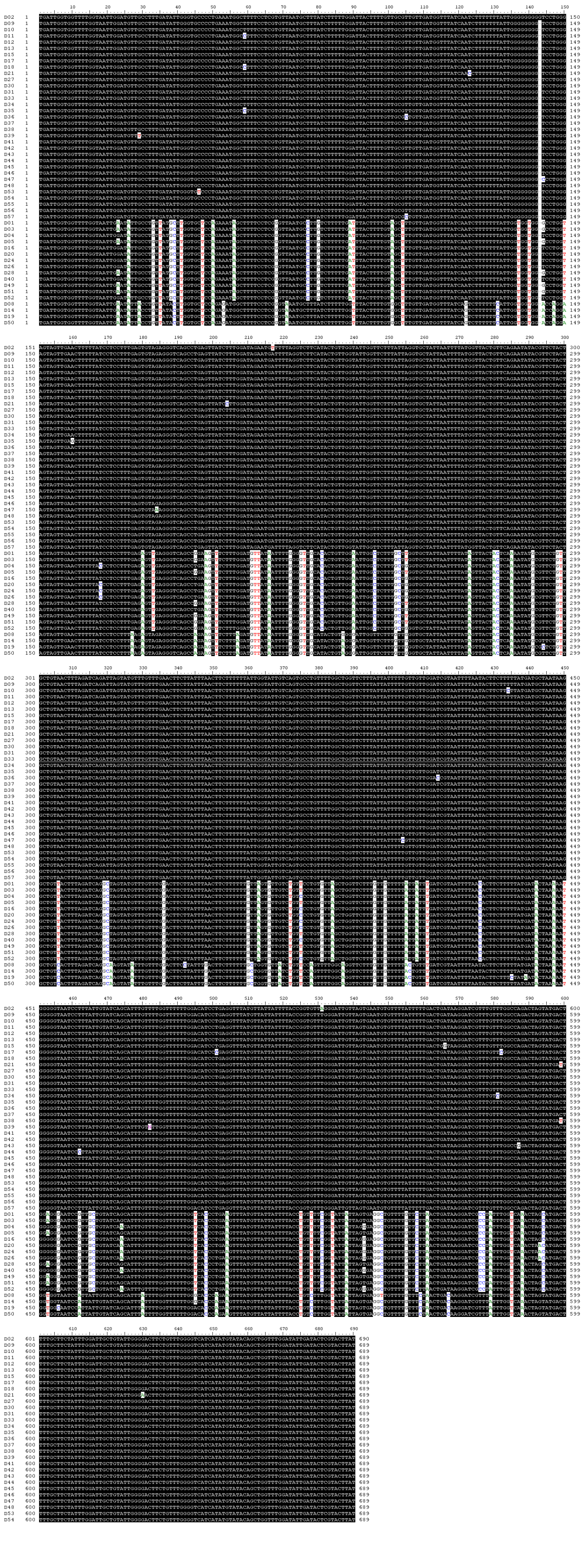

Figure S3: Sequence alignment of the filaria nematode based on the partial *COI* gene.

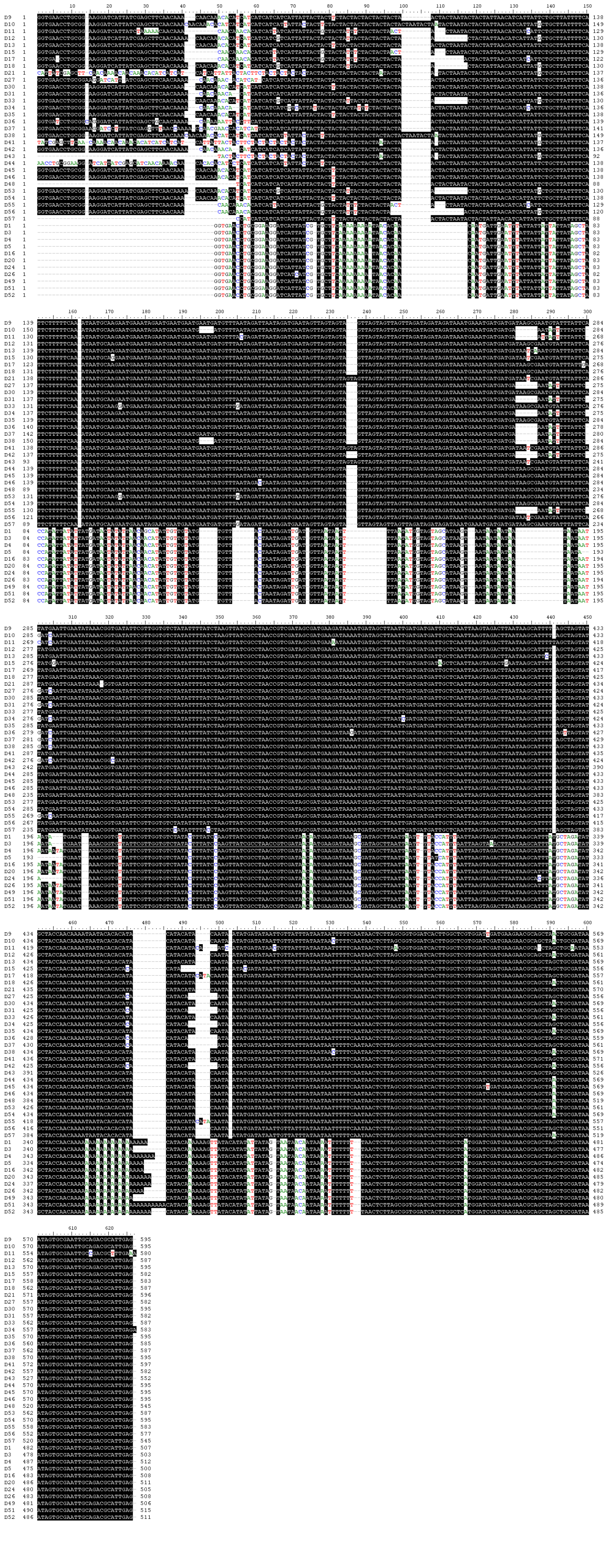

Figure S4: Sequence alignment of the filaria nematode based on the *ITS1* region

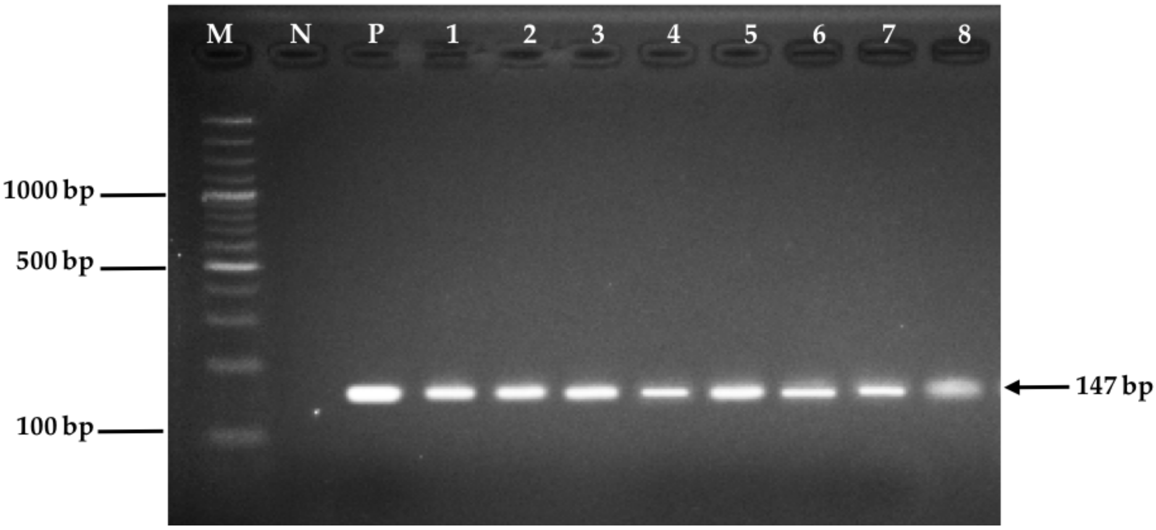

Figure S5: PCR amplicons of the *FtsZ* specific to *Wolbachia*. Lane M: molecular mass marker (100 base pairs [bp]). Lane P1: *Wolbachia* positive control. Lane N: Negative control. Lanes 1–8: Positive samples.

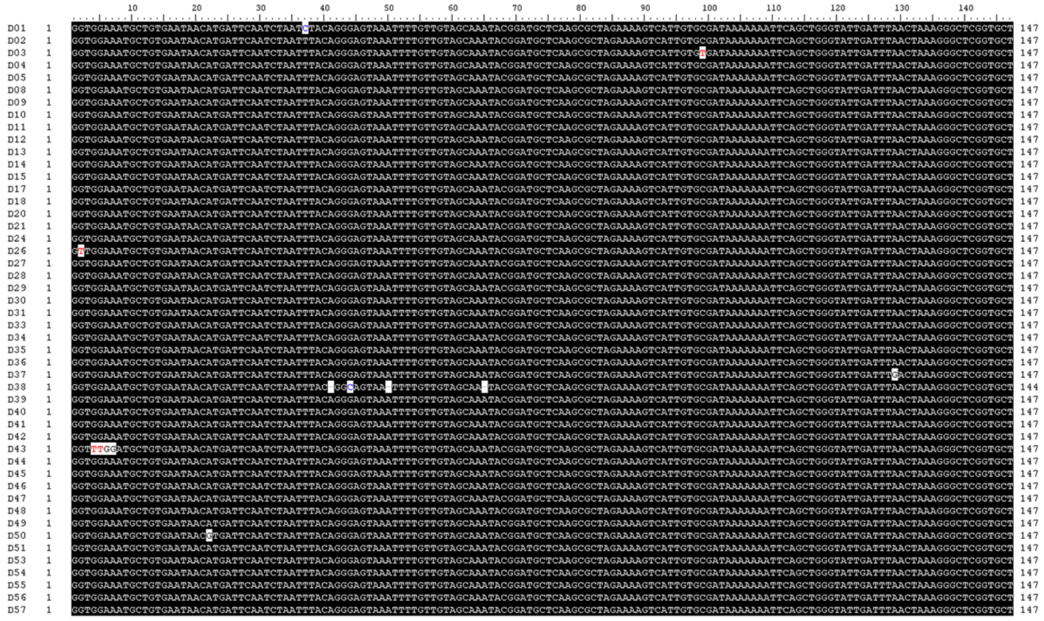

Figure S6: Sequence alignment of *Wolbachia* bacteria based on the *FtsZ* gene.

Table S1: Raw data of samples.

CODE	COI gene	ITS1 region	FtsZ gene
D01	*B. pahangi*	*B. pahangi*	*Wolbachia*
D02	*D. immitis*	-	*Wolbachia*
D03	*B. pahangi*	*B. pahangi*	*Wolbachia*
D04	*B. pahangi*	*B. pahangi*	*Wolbachia*
D05	*B. pahangi*	*B. pahangi*	*Wolbachia*
D06	*B. malayi*	-	*Wolbachia*
D07	-	-	-
D08	-	-	-
D09	*D. immitis*	*D. immitis*	*Wolbachia*
D10	*D. immitis*	*D. immitis*	*Wolbachia*
D11	*D. immitis*	*D. immitis*	*Wolbachia*
D12	*D. immitis*	*D. immitis*	*Wolbachia*
D13	*D. immitis*	*D. immitis*	*Wolbachia*
D14	*B. malayi*	-	*Wolbachia*
D15	*D. immitis*	*D. immitis*	*Wolbachia*
D16	*B. pahangi*	*B. pahangi*	-
D17	*D. immitis*	*D. immitis*	*Wolbachia*
D18	*D. immitis*	*D. immitis*	*Wolbachia*
D19	-	-	-
D20	*B. pahangi*	*B. pahangi*	*Wolbachia*
D21	*D. immitis*	*D. immitis*	*Wolbachia*
D22	-	-	-
D23	-	-	-
D24	*B. pahangi*	*B. pahangi*	*Wolbachia*
D25	-	-	-
D26	*B. pahangi*	*B. pahangi*	*Wolbachia*
D27	*D. immitis*	*D. immitis*	*Wolbachia*
D28	*B. pahangi*	-	*Wolbachia*

D29	*B. malayi*	*B. pahangi*	*Wolbachia*
D30	*D. immitis*	*D. immitis*	*Wolbachia*
D31	*D. immitis*	*D. immitis*	*Wolbachia*
D32	-	-	-
D33	*D. immitis*	*D. immitis*	*Wolbachia*
D34	*D. immitis*	*D. immitis*	*Wolbachia*
D35	*D. immitis*	*D. immitis*	*Wolbachia*
D36	*D. immitis*	*D. immitis*	*Wolbachia*
D37	*D. immitis*	*D. immitis*	*Wolbachia*
D38	*D. immitis*	*D. immitis*	*Wolbachia*
D39	*D. immitis*	-	*Wolbachia*
D40	*B. pahangi*	-	*Wolbachia*
D41	*D. immitis*	*D. immitis*	*Wolbachia*
D42	*D. immitis*	*D. immitis*	*Wolbachia*
D43	*D. immitis*	*D. immitis*	*Wolbachia*
D44	*D. immitis*	*D. immitis*	*Wolbachia*
D45	*D. immitis*	*D. immitis*	*Wolbachia*
D46	*D. immitis*	*D. immitis*	*Wolbachia*
D47	*D. immitis*	-	*Wolbachia*
D48	*D. immitis*	*D. immitis*	*Wolbachia*
D49	*B. pahangi*	*B. pahangi*	*Wolbachia*
D50	*B. malayi*	-	*Wolbachia*
D51	*B. pahangi*	*B. pahangi*	*Wolbachia*
D52	*B. pahangi*	*B. pahangi*	-
D53	*D. immitis*	*D. immitis*	*Wolbachia*
D54	*D. immitis*	*D. immitis*	*Wolbachia*
D55	*D. immitis*	*D. immitis*	*Wolbachia*
D56	*D. immitis*	*D. immitis*	*Wolbachia*
D57	*D. immitis*	*D. immitis*	*Wolbachia*
