# Supplementary figures and images for: Molecular Analysis of Canine Filaria and Its Wolbachia Endosymbionts in Domestic Dogs Collected from Two Animal University Hospitals in Bangkok Metropolitan Region, Thailand
Source: Pathogens. 2019 Jul 29;8(3):114. doi: 10.3390/pathogens8030114 (PMC6789508; doi:10.3390/pathogens8030114)

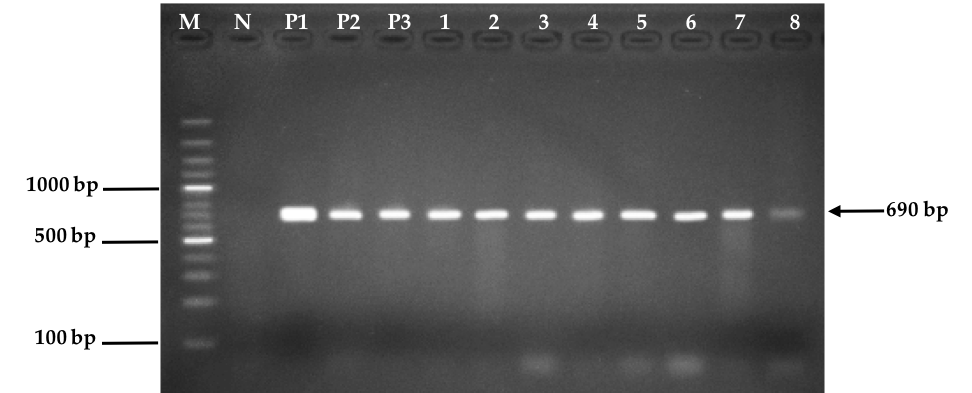

Supplement: Supplementary file 1 [file pathogens-08-00114-s001.zip › Figure supplement/Figure S1.png]

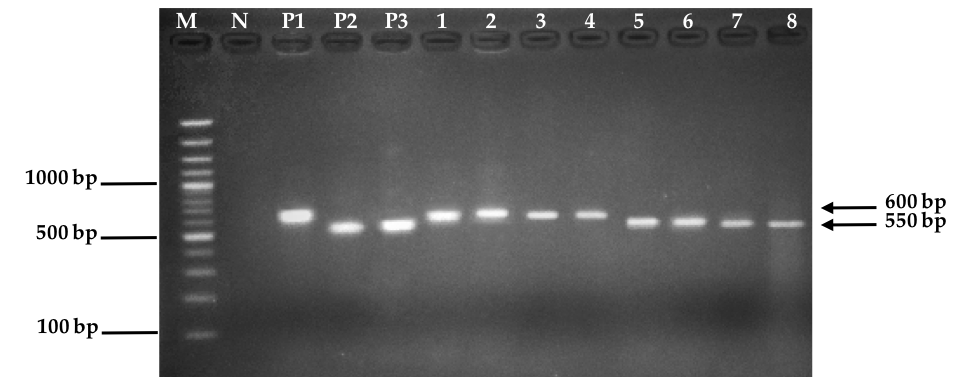

Supplement: Supplementary file 1 [file pathogens-08-00114-s001.zip › Figure supplement/Figure S2.png]

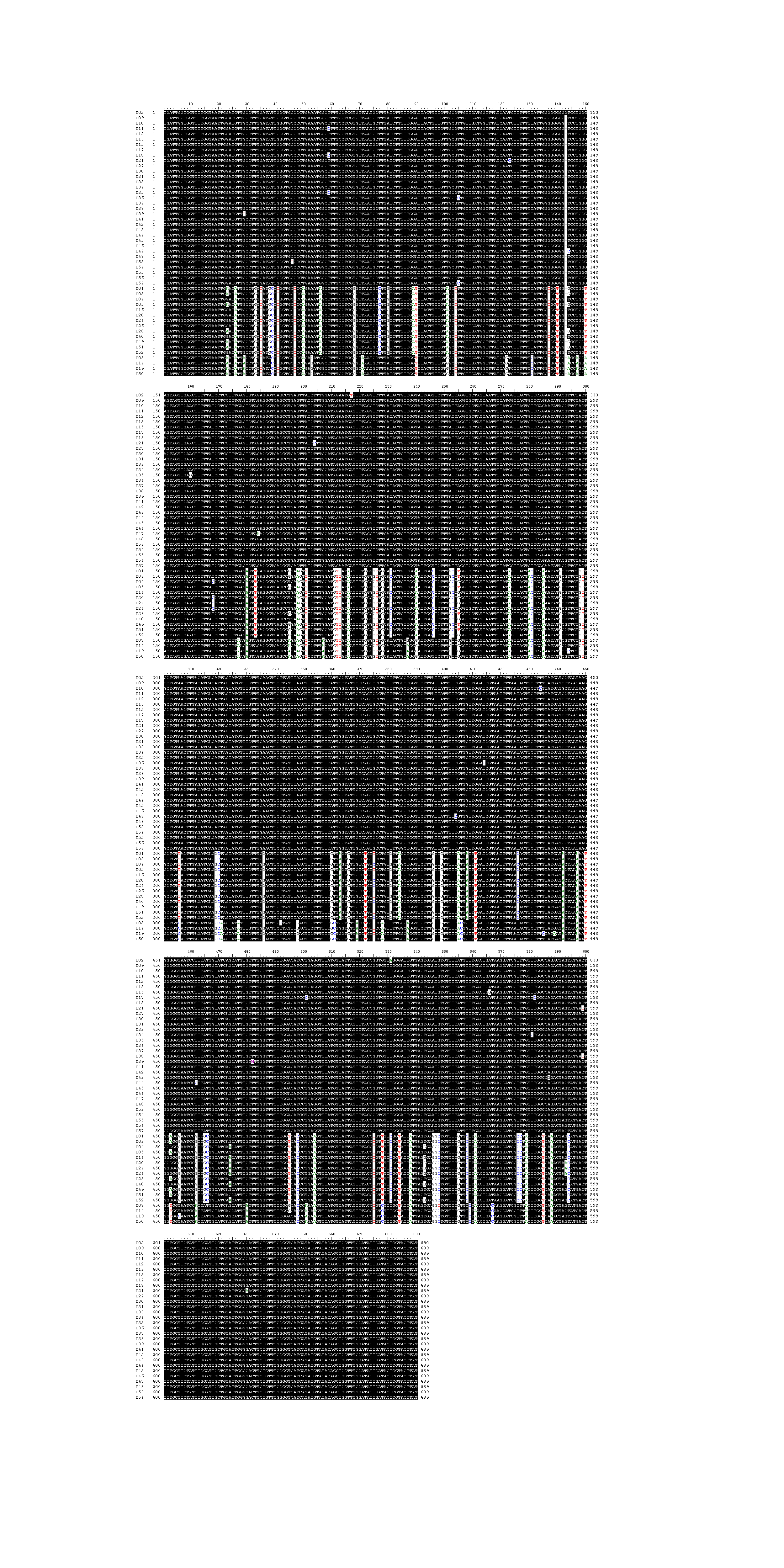

Supplement: Supplementary file 1 [file pathogens-08-00114-s001.zip › Figure supplement/Figure S3.png]

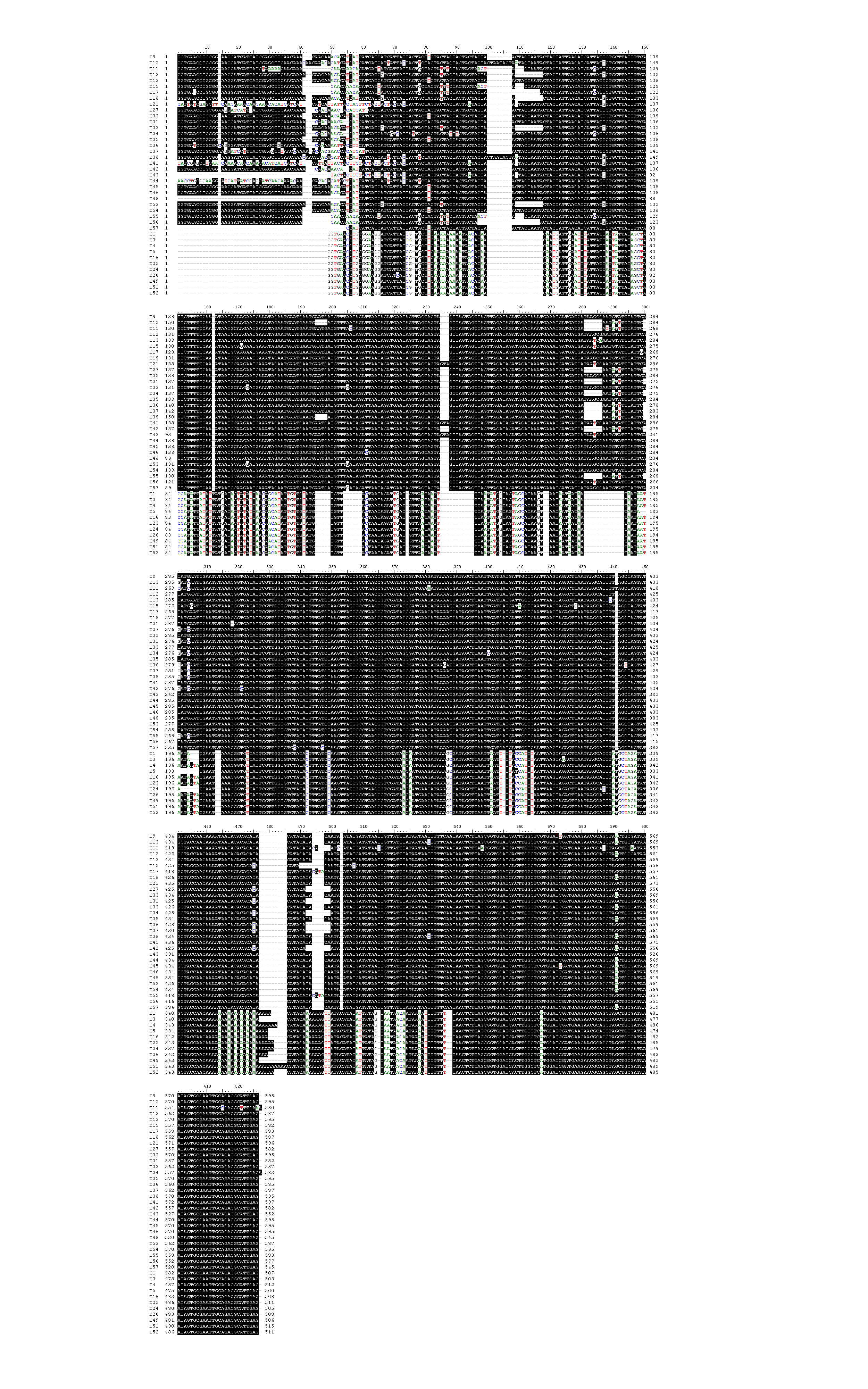

Supplement: Supplementary file 1 [file pathogens-08-00114-s001.zip › Figure supplement/Figure S4.png]

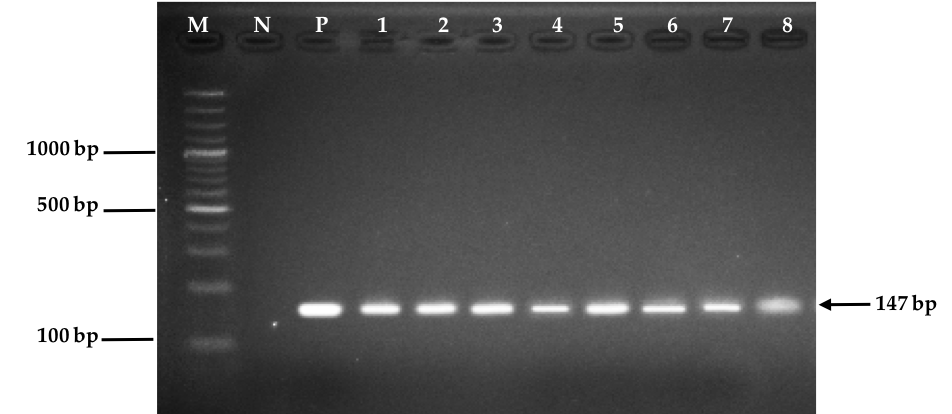

Supplement: Supplementary file 1 [file pathogens-08-00114-s001.zip › Figure supplement/Figure S5.png]

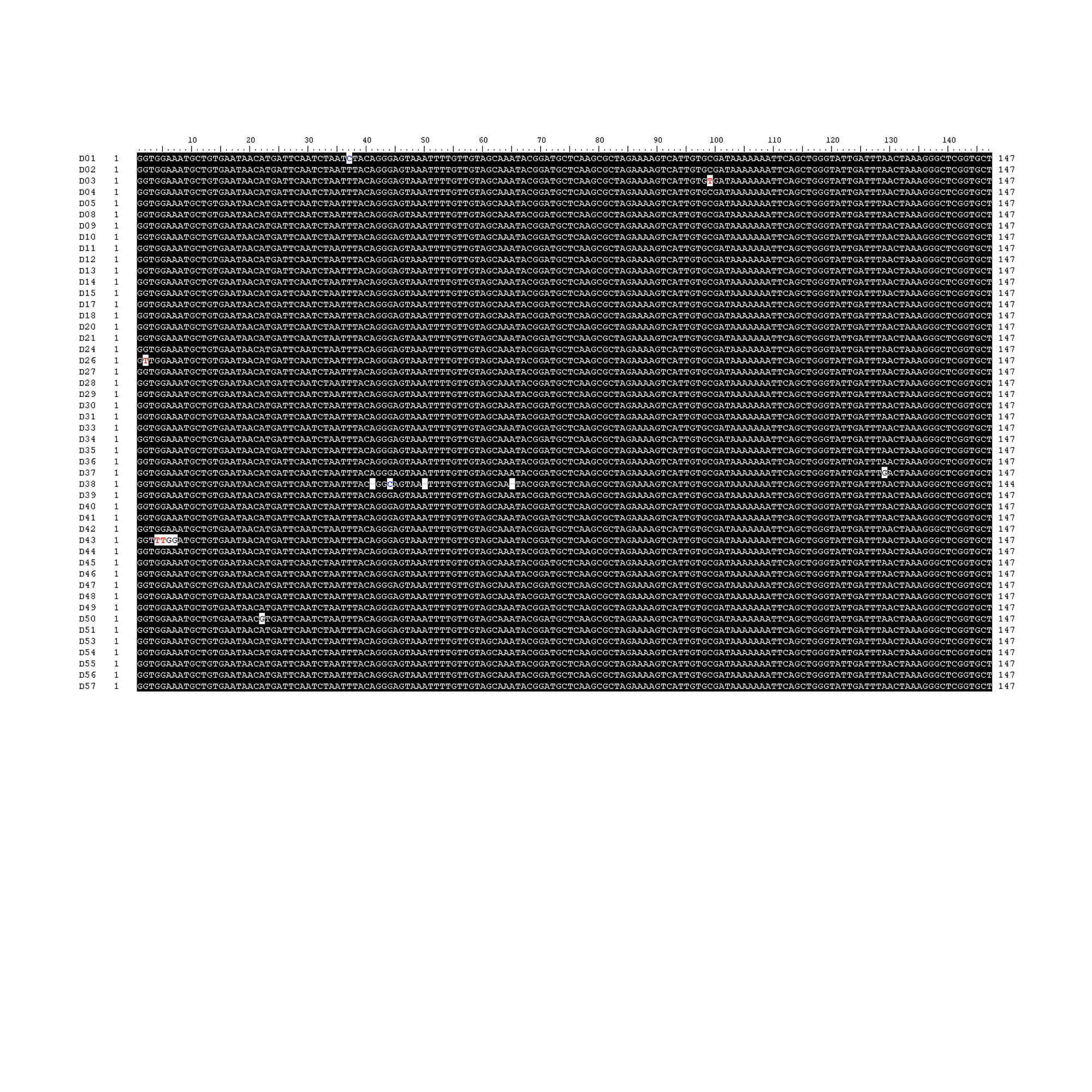

Supplement: Supplementary file 1 [file pathogens-08-00114-s001.zip › Figure supplement/Figure S6.png]
